# Ileus Caused by Compression of the Small Intestine in 35 Cows

**DOI:** 10.3390/ani15040569

**Published:** 2025-02-16

**Authors:** Ueli Braun, Christian Gerspach, Elena Bennien, Monika Hilbe, Karl Nuss

**Affiliations:** 1 Department of Farm Animals, Vetsuisse Faculty, University of Zurich, 8057 Zurich, Switzerland; cgerspach@vetclinics.uzh.ch (C.G.); elenabennien@icloud.com (E.B.); karl.nuss@uzh.ch (K.N.); 2 Institute of Veterinary Pathology, Vetsuisse Faculty, University of Zurich, 8057 Zurich, Switzerland; hilbe@vetpath.uzh.ch

**Keywords:** cattle, small intestine, ileus, compression

## Abstract

Ileus attributable to compression of the small intestine describes extensive constriction of the intestine by nearby space-occupying lesions or other abdominal organs. This retrospective study describes the findings in 35 cows with ileus caused by CSI (compression of the small intestine) by adhesions, abscesses, and the gravid uterus. Colic occurred in 34.3% of the cows. Rumen motility was reduced or absent in all cows, and intestinal motility was decreased in 85.7%. Ballottement and/or percussion and simultaneous auscultation were positive on the right side in 73.5% of the cows. The rectum contained little or no faeces in all cows, and transrectal examination revealed dilated small intestine in 25.7% of the cows, but the actual site of compression could not be palpated. The main ultrasonographic findings were reduced or absent intestinal motility and dilated loops of small intestine. Thirty-one of the thirty-five cows underwent right flank laparotomy; fourteen of these were euthanised intraoperatively, and seventeen recovered and were subsequently discharged. CSI is a rare cause of ileus. Improvements in the diagnosis of CSI are needed to prevent unnecessary laparotomy in cows with a grave prognosis.

## Simple Summary

Ileus (Latinisation of the Greek *εἰλεός ileós*) is the interruption of the passage of intestinal contents (intestinal obstruction). Ileus can be either functional (paralytic) or mechanical; the latter is most common in cattle and can be caused by intussusception, strangulation, incarceration, volvulus, mesenteric torsion, and compression of the small intestine. Ileus attributable to compression of the small intestine (CSI) describes extensive constriction of the intestine by nearby space-occupying lesions or other abdominal organs. This retrospective study describes the findings, treatment, and outcome in 35 cows with ileus caused by CSI by adhesions, abscesses, and the gravid uterus. CSI can rarely be diagnosed without surgery because the clinical, laboratory, and ultrasonographic findings of the different forms of ileus overlap. However, a correct diagnosis is important since the prognoses of the different forms of ileus vary greatly. In cows with CSI, the prognosis depends primarily on the cause of the compression and is much better in cows with compression caused by adhesions or the gravid uterus compared with an abscess. Thirty-one of the thirty-five cows underwent right flank laparotomy; fourteen of these were euthanised intraoperatively, and seventeen recovered and were subsequently discharged.

## 1. Introduction

Compression of the small intestine (CSI) describes a two-dimensional constriction of the small intestine by the gravid uterus or nearby space-occupying lesions, including abscesses, fat necrosis, tumours, haematomas [1], and other abnormalities [2,3,4,5,6,7]. One report described eight cows with compression of the duodenum caused by adhesions associated with a liver abscess [3], followed by two other cases of duodenal and jejunal compression by liver abscesses [4]. Abscesses caused by right flank intraperitoneal injection can compress adjacent sections of the descending part of the duodenum [2]. Constrictive, fibrous, or fibrinous adhesions attributable to inflammation are also considered important causes of CSI [2,4,7]; these can arise from laparotomy, omento- or pyloropexy, peritonitis, and perforating abomasal and intestinal ulcers [7]. Adhesions at the duodenal sigmoid flexure were identified as the cause of CSI in 14 Holstein cows [7]; in all of those cases, the ascending duodenum was dilated and the descending duodenum was empty. Eight of the fourteen cows had undergone an omentopexy to correct right displaced abomasum or duodenal volvulus, which was assumed to be the cause of the adhesions. Inflammatory lesions may be associated with adhesions of the small intestine after accidental puncture of the dorsal cranial vagina and intraperitoneal infusion of irritating intrauterine drugs. The result is often chemical or bacteriological peritonitis leading to the formation of extensive adhesions or abscesses, which can involve the small intestine [6]. Loops of the small intestine may be trapped and compressed between the pelvis and the foetus during parturition [6]. The gravid uterus near the end of gestation [7] has also been implicated in CSI. The same is true for a displaced and/or dilated gallbladder [4,5]. Haemorrhage after enucleation of the corpus luteum has become a less likely cause of CSI [8] because luteolysis can be achieved reliably using prostaglandin F_2α_ [9].

The most frequent clinical signs in cows with duodenal compression by a liver abscess were a moderately to severely abnormal demeanour, severe impairment of gastrointestinal function, and reduced or absent faecal output [3]. In 14 cows with adhesions involving the sigmoid flexure of the duodenum, the clinical signs were vague and included dullness, inappetence, absence of faecal output, increases in heart and respiratory rates, and dehydration [7]. Cows with CSI attributable to fat necrosis have an insidious onset of illness characterised by weight loss, decreased faecal output, abdominal distension, and mild colic [2,6,10]. Firm masses of varying size may be palpated in the abdominal cavity of these cattle during transrectal palpation [1,11].

In cows with CSI, ultrasonography is primarily used to visualise the typical characteristics of small intestinal ileus, which are dilated loops of small intestines with a diameter of ≥4 cm and reduced or absent intestinal motility [12]; however, the actual site of compression is rarely seen. The site of compression was seen in only one of 35 cows with duodenal compression by a liver abscess and in one other cow in which the duodenum was compressed between the liver and dilated gallbladder [4]. In another cow, compression of the duodenum by a displaced gallbladder could be seen ultrasonographically [5]. Ultrasonographic visualisation of the site of compression has also been reported in cows with fat necrosis [11].

The response to treatment of CSI depends primarily on the cause of the compression. The standard treatment for CSI involves right flank laparotomy [6,13,14,15]. Surgical treatment of cows with CSI caused by abscesses, tumours, or fat necrosis is usually not successful [1]. Two other studies reported that all cows with CSI caused by liver abscess had to be slaughtered or euthanised [3,4]. In contrast, 13 of 14 cows that underwent side-to-side anastomosis of the cranial and descending duodenum because of adhesions at the duodenal sigmoid flexure survived and were discharged [7]. In one cow with compression of the duodenum between the liver and the severely dilated gallbladder, the latter had to be drained to facilitate repositioning of the duodenum [4]. In an 11-month-old Holstein heifer with duodenal compression by a displaced gallbladder, the latter was repositioned, and when the condition recurred 4 days later, anatomical fixation of the gallbladder was carried out [5]. With a few exceptions, the published cases of CSI in cattle involved the duodenum. To our knowledge, a large case series of CSI has not been performed. Furthermore, some clinicians may not be as familiar with CSI as with other types of ileus. Volvulus, strangulation, and intussusception are commonly dealt with in textbooks, whereas CSI is not often addressed specifically. Therefore, the goal of this study was to focus on CSI by describing its progression in 35 cows.

## 2. Materials and Methods

The medical records of 35 cows diagnosed with CSI between 1986 and 2016 at the Department of Farm Animals, University of Zurich, were analysed in a dissertation [16].

### 2.1. Definitions Used

The term CSI was used for cattle with mechanical ileus due to compression of the small intestine by nearby space-occupying lesions or other abdominal organs.

Abomasal reflux syndrome was diagnosed in cattle that had hypochloraemic, hypokalaemic, metabolic alkalosis (chloride ≤ 95 mmol/L, potassium < 4 mmol/L, bicarbonate > 30 mmol/L, base excess > 2 mmol/L) with or without an increase in rumen chloride concentration (rumen chloride > 30 mmol/L) [17].

A final diagnosis of CSI (for the retrospective analysis of the medical records) was made during laparotomy and/or postmortem examination.

A surviving animal was defined as a patient discharged from the clinic.

### 2.2. Inclusion and Exclusion Criteria

Included animals were a minimum of one year of age and had CSI at the time of admission. Seven cows in the dissertation [16] were excluded because they did not fulfil the definition of CSI outlined in the Introduction. Another cow with compression of the duodenum between the liver and gallbladder and also from the dissertation was excluded because the findings were published previously [4].

### 2.3. Clinical Examination

All cows underwent a standard clinical examination [18,19]. The general condition was evaluated by determining the demeanour, behaviour, posture (including recumbency), appetite, signs of abdominal pain, appearance of the hair coat and muzzle, skin elasticity, position of the eyes in the sockets, and skin surface temperature. General condition was classified as normal or mildly, moderately, or severely abnormal. The general condition was considered mildly abnormal when a mild decrease in alertness and/or mild signs of colic (defined below) were present. A moderate decrease in alertness and sometimes occasional grunting, and/or bruxism and marked signs of colic were observed in cattle with a moderately abnormal general condition. Cattle with a severely abnormal general condition showed marked apathy and were sometimes recumbent and unable to rise.

Each cow was observed for signs of pain, which were judged as mild, moderate, or severe, as described [19,20]. Signs of mild colic included mild restlessness, shifting of weight in the hind limbs, looking at the flank, lifting the tail, lifting of individual limbs, and tail swishing. Signs of moderate colic were moderate restlessness, brief periods of recumbency, kicking with the hind limbs, arching of the back, and marked tail swishing. Signs of severe colic consisted of marked restlessness, frequent lying down and rising, sweating, grunting, and violent kicking at the abdomen.

In addition, the cattle were divided into colic (see above), indolence (dullness), and intoxication phases, depending on the stage of illness [19,20]. The indolence phase followed the colic phase and was characterised by apathy and a markedly abnormal general condition. The last phase was intoxication in which cattle had tachycardia, congested scleral blood vessels, pale mucous membranes, cool skin surface temperature, sunken eyes and a dry muzzle.

### 2.4. Laboratory Analyses

The collection and examination of blood, urine, and rumen fluid were performed as described [17]. The following blood samples were collected from the left jugular vein: 5 mL of EDTA blood for haematological analysis, 10 mL of whole blood for serum biochemistry, and 2 mL of whole blood mixed with 0.2 mL heparin for venous blood gas analysis. Haematological analysis included the determination of haematocrit, total leukocyte count, and the concentrations of total protein and fibrinogen. The samples were analysed using the Contraves analyser AL820 (Contraves, Oerlikon, Switzerland) or the CELL-DYN 3500 (Abbott Diagnostics Division, Baar, Switzerland). The concentrations of urea, bilirubin, calcium, magnesium, inorganic phosphate, chloride, potassium, and the activities of the enzymes aspartate aminotransferase (AST) and γ-glutamyltransferase (γ-GT) were determined at 37 °C using an automated analyser (Cobas Mira, Cobas Integra 700, Cobas Integra 800, Roche Diagnostics, Basel, Switzerland) and the manufacturer’s reagents (Roche-Reagents) according to the International Federation of Clinical Chemistry and Laboratory Medicine (IFCC). The venous blood gas analysis was performed with the RapidLab 248 analyser (Siemens Schweiz AG, Zurich, Switzerland). Urine samples were collected by free-flow or catheterisation and analysed using a test strip (Combur9, Roche, Basel, Switzerland). A refractometer (Krüss Optronic, Hamburg, Germany) was used to measure specific gravity. A sample of rumen fluid was collected using a Dirksen probe and assessed for colour, odour, consistency, and pH. In addition, the concentration of chloride was determined (MK-II-Chlorid-Analyser 9265, Sherwood, Cambridge, Great Britain).

### 2.5. Ultrasonographic Examination of the Abdomen

The abdomen of 23 cows was scanned from the right side, as described [12]. Briefly, the area from the tuber coxae to the eighth intercostal space and from the transverse processes of the vertebrae to the linea alba on the right side was examined using a 3.5 to 6.5 MHz convex transducer (GE Logiq 7, scil animal care company GmbH, Viernheim, DE) after clipping the hair and applying contact gel to the transducer and alcohol to the clipped skin. The appearance of loops of small intestine and their diameter, contents and motility were assessed. In addition, the appearance, position and nature of the contents of the caecum and proximal and spiral ansa of the colon and the presence of caecal dilatation were noted [12]. Abomasal dilatation was diagnosed when the abomasum was subjectively dilated and filled with hypoechogenic ingesta and fluid. In cattle with a dilated abomasum, the abomasal folds appeared as thin, echogenic, wavy structures. Fibrin appeared as irregularly shaped echogenic masses or strands between the intestinal loops.

### 2.6. Diagnosis

A diagnosis of ileus based on the clinical examination was made when the main findings were characteristic of mechanical ileus, which included signs of colic, reduced, or absent faecal output and progressive deterioration in demeanour [21]. An ultrasonographic diagnosis of ileus was made when dilated loops of small intestine with a diameter of ≥4.0 cm were seen accompanied by severely reduced or absent intestinal motility in ultrasonograms. A diagnosis of ileus attributable to CSI was made when a compression and dilated small intestine proximal to the compression site and empty intestine distal to the compression site were seen. A final diagnosis of CSI was made during laparotomy and/or postmortem examination based on the occurrence of a small intestine that was compressed by adhesions, enlarged organs (pregnant uterus), or space-occupying lesions (abscess), and dilated proximal to the lesion and empty distal to the lesion.

### 2.7. Laparotomy and Postoperative Treatment

Twenty-eight cows were operated immediately after admission. Of the remaining three cows, one (surviving) was operated one day and the other two (non-surviving) three and four days later. Thirty-one cows underwent standing right flank laparotomy [22]. In one cow that went down during surgery, the operation was finished with the cow in sternal recumbency. Distal paravertebral anaesthesia was mainly used until 2001, after which time proximal paravertebral anaesthesia was used. A 25 to 30 cm incision was made in the mid-paralumbar fossa. After routine abdominal exploration, the section of small intestine that was dilated was exteriorised and examined when possible. Adhesions involved in the CSI were broken down manually whenever possible but when this was not feasible or the intestinal lesions were severe, intestinal resection with end-to-end anastomosis was carried out [23]. After the surgical procedure, an antibiotic, most commonly amoxicillin (Clamoxyl, Zoetis Schweiz, Delémont, Switzerland, 7 mg/kg body weight), was administered intra-abdominally in 1 L of isotonic saline solution or polyvinylpyrrolidone (before its use in farm animals was discontinued in Switzerland). The peritoneum and the muscle layers were closed with synthetic, absorbable, lactomer polyfilamentous suture material (Polysorb TM, USP 2, Medtronic, Dublin, Ireland) using a simple continuous suture pattern. A modified mattress suture pattern was used to close the subcutaneous tissues (Lactomer, USP 0, Medtronic), and skin staples (Covidien Appose Single Use Surgical Skin Stapler, size 35 w, Medtronic) were used to close the skin.

The cows that were successfully operated and subsequently discharged received fluid therapy, antibiotics, analgesics, prokinetic drugs, and electrolyte replacement (for details, see Section 3.8.

### 2.8. Euthanasia/Slaughter and Postmortem Examination

Cows were euthanised using pentobarbital (80 mg/kg body weight, administered intravenously, Esconarkon, Streuli Pharma, Uznach, Switzerland) or, in earlier years, they were sent to the slaughter facility of the Veterinary Hospital during or after the initial examination when they were in serious clinical condition [19,20] or when the owner did not consent to surgery. The meat of slaughtered cattle was used for zoo-animal feeding when this was still permitted. Cattle were euthanised intraoperatively when lesions associated with a very poor prognosis were seen or complications occurred or postoperatively when the clinical condition deteriorated. All cattle that died or were euthanised underwent postmortem examination, whereas in slaughtered cattle, only the internal organs were inspected.

### 2.9. Statistics

The program SPSS Statistics 26.0 (IBM Switzerland, Zurich, Switzerland) was used for analysis. Frequencies were determined for all variables, and the Shapiro–Wilk test was used to test the numerical data for normality. Means ± standard deviations were calculated for normal data and medians for non-normal data. In addition, the 95% CI was calculated for the means and medians, respectively. Differences between the numerical data of cows with CSI due to adhesions, abscesses, and pregnant uterus were analysed using the Kruskal–Wallis test. Differences in nominal data were analysed using the chi-square test, and if a sample number was <10, Fisher’s exact test was used. When the *p*-value was <0.05, a post hoc test (Bonferroni correction) was used to identify groups that differed significantly. The variables heart rate and rectal temperature over time (days 0 to 7, day 0 = day of admission, day 7 = day 7 post laparotomy) were analysed using the general linear model, choosing ANOVA with repeated measures and replacing polynomial contrasts with difference. A value of *p* < 0.05 was considered significant.

## 3. Results

### 3.1. Cattle and History

There were 34 (97.1%) cows and 1 (2.9%) heifer, ranging in age from 2.3 to 12.0 years (median, 95% CI, 4.0, 3.5–5.0 years). The breeds included 26 (74.3%) Swiss Braunvieh, 4 (11.4%) Holstein, 4 Swiss Fleckvieh, and 1 (2.9%) Jersey cow. Nineteen (54.3%) were pregnant between 9 and 41 weeks (mean ± sd, 95% CI, 24.9 ± 10.4, 20–30 weeks), twelve (34.3%) were open, and the pregnancy status (pregnant or open) was not recorded in the remaining four (11.4%). The date of the last calving was known in 14 cows and was 0.4 to 28 weeks before admission to our clinic (median, 95% CI, 6.3, 1–8 weeks). The duration of illness of all cows before admission ranged from 8 h to 14 days (median, 95% CI, 48, 24–72 h). Twenty-two cows (62.9%) were anorexic, and thirteen (37.1%) had a reduced appetite. Eighteen (51.4%) had a history of colic before admission.

### 3.2. Clinical Findings

The general condition was mildly abnormal in 25.7% (9/35), moderately abnormal in 57.1% (20/35), and severely abnormal in 17.1% (6/35) of the cows. Ten cows (28.6%) had unilateral or bilateral abdominal dilatation, and 14.3% (5/35) had nonspecific signs of pain, including muscle tremors (2/35, 5.7%), piloerection (2/35, 5.6%), or bruxism (1/35, 2.9%). Twelve (34.3%) cows were in the colic phase, but detailed information was only available for seven. Colic manifested as treading (7/35, 20.0%), lordosis (3/35, 8.6%), and kicking (2/35, 5.7%). Six (17.1%) cows had one and one cow (2.9%) had two visceral signs of pain, and the colic was assessed as mild (10/35, 28.6%) or moderate (2/35, 5.7%). Twenty (57.1%) of the cows were in the indolence (dullness) phase and three (8.6%) were in the intoxication phase at the time of admission. In addition, 60.0% (21/35) of the cows had a tense abdominal wall.

Tachycardia occurred in 42.9% (15/35), decreased rectal temperature in 42.9% (15/35) and tachypnoea in 42.9% (15/35) of the cows (Table 1).

The most frequent abnormalities were reduced or absent rumen motility (100%, 35/35), little or no faecal output (100%, 35/35), reduced or absent intestinal motility (85.7%, 30/35), and positive ballottement and/or percussion and simultaneous auscultation on the right side (73.5%, 25/34). Transrectal palpation revealed rumen dilatation in 10 (28.6%) and dilation of the small intestine in 9 (25.7%) cows (Table 1). The actual cause of the compression could not be palpated in any of the cows. Other abnormal findings included a positive result in at least one of three foreign body tests in 25.7% (9/35) of the cows and mild to moderate rumen tympany in 5.7% (2/35). The faeces were dark brown to black in 5.7% (2/35) of the cows and the consistency varied from liquid to pulpy (normal) to thick pulpy and pasty. Abnormal faecal contents occurred in 45.7% (16/35) of the cows and included mucus, blood, or fibrin.

Other clinical abnormalities were reduced skin surface temperature (71.4%, 25/35), reduced skin elasticity (62.9%, 22/35), moderately to severely hyperaemic scleral vessels (62.9%, 22/35), prolonged capillary refill time (52.9%, 18/34), sunken eyes (51.4%, 18/35), dry and cool muzzle (31.4%, 11/35), ammonia-like or otherwise foul breath (22.9%, 8/35), and pale oral mucous membranes (11.4%, 4/35).

### 3.3. Urinalysis and Laboratory Findings

Urine pH ranged from 5.0 to 9.0 (median, 95% CI, 7.0, 6.0–8.0) and was acidic (5.0–6.9) in 38.2% (13/34) of cows and alkaline (8.1–9.0) in 17.6% (6/34). Specific gravity was decreased (<1.020) in 41.9% (13/31) of the cows. Glucosuria occurred in 32.4% (11/34), haemoglobinuria/haematuria in 26.9% (9/34), ketonuria in 14.7% (5/34), and proteinuria in 5.9% (2/34) of the cows.

The principal abnormalities were hypokalaemia (82.9%, 29/35), positive base excess (80.0%, 24/30), hypermagnesaemia (75.0%, 15/20), hypocalcaemia (68.2%, 15/22), hypochloraemia (64.7%, 22/34), hypercapnia (63.4%, 19/30), azotaemia (62.9%, 22/35), hyperproteinaemia (62.9%, 22/35), hyperfibrinogenaemia (57.1%, 20/35) and increased concentration of blood bicarbonate (53.3%, 16/30) (Table 2). Less common changes included haemoconcentration (45.7%, 16/35), alkalosis (46.7%, 13/30), leukocytosis (42.8%, 15/35), hyperbilirubinaemia (34.3%, 12/35), increased rumen chloride concentration (29.4%, 10/34), increased activities of aspartate aminotransferase (25.7%, 9/35) and gamma-glutamyl transferase (20.0%, 7/35), and hypophosphataemia (14.3%, 3/21).

### 3.4. Ultrasonographic Findings

The principal findings were subjectively reduced or absent intestinal motility (100%, 20/20) and dilated loops of small intestine (91.3%, 21/23) (Table 3). Abdominal fluid with or without fibrin was seen in 39.1% (9/23) of the cows. Empty poststenotic loops of small intestine were seen in 26.1% (6/23) of the cows. Compression caused by a multi-chambered abdominal abscess with adhesions involving the jejunum could be seen in one cow (no. 35, 4.3%). In 21.7% (5/23) of the cows, the abomasum had retrograde impaction and was therefore dilated.

### 3.5. Concomitant Diseases

Eight of 35 (22.9%) cows had CSI together with one or two comorbidities (fatty liver syndrome, abomasal ulcer, gastrointestinal nematodes, dicrocoeliosis). The comorbidities were in all likelihood not related to CSI.

### 3.6. Treatment and Outcome

**Figure 1:**
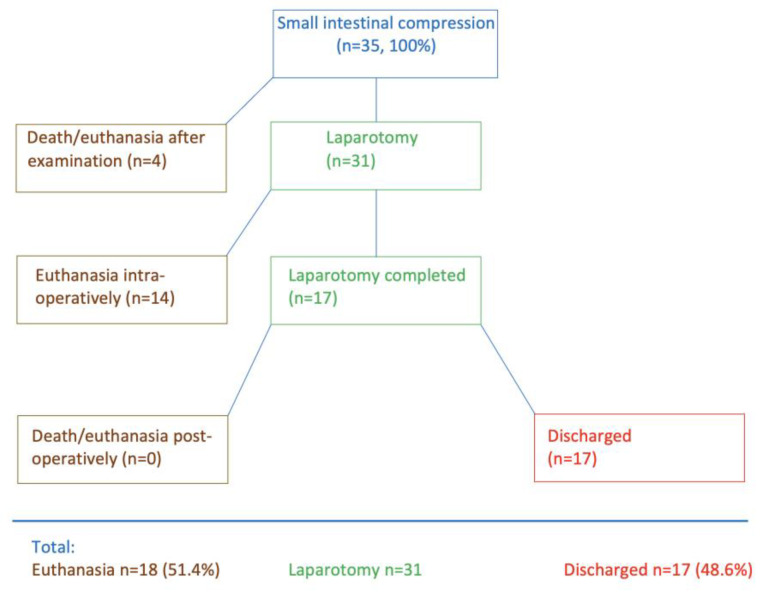
Treatment flowchart for 35 cattle with CSI.

Four of the 35 cows died immediately after the initial examination or had to be euthanised (Figure 1) because of severe illness, a grave prognosis, or because the owner did not consent to surgery. Thirty-one cows underwent right flank laparotomy; fourteen of these were euthanised during surgery, and seventeen were discharged after successful postoperative treatment. Thus, 51.4% (18/35) died and 48.6% (17/35) survived.

### 3.7. Surgical Findings, Complications, and Postmortem Findings in 4 Slaughtered or Euthanised Cows After Examination and in 31 Cows Treated Surgically

The CSI involved the duodenum in 17.1% (6/35), the jejunum in 74.3% (26/35), the duodenum and the jejunum in 2.9% (1/35) and the jejunum and the ileum in 5.7% (2/35) of the cases (these numbers include the 4 non-operated cows, in which the diagnosis was confirmed during postmortem examination).

The causes of CSI were adhesions (*n* = 16), abscesses (*n* = 15), and the pregnant late-term uterus (*n* = 4). However, most surviving cows had either adhesions or compression by the gravid uterus, while most non-surviving cows had an abscess compressing the small intestine. The cause of CSI in the 4 non-operated cows (no. 3, 11, 17, 35) was an abscess. In the 31 operated cows, the cause of CSI was adhesions in 16 cows, abscesses in 11 cows, and the pregnant uterus in 4 cows. Of the 17 surviving cows (Table 4), 12 had CSI caused by adhesions between the small intestine and the uterus (no. 12), urinary bladder (no. 10), omasum (no. 6), greater or lesser omentum (no. 15, 18, 31), another small intestinal loop (no. 33), liver (no. 23, 26, 30), abdominal wall (no. 20), and the omentopexy site after abomasal surgery (no. 27). The adhesions were broken down manually (*n* = 7) or resolved by removing the omentopexy (*n* = 1), or surgically via intestinal resection (end-to-end anastomosis, no. 12, 15, 20, 33). In two cows that were near the end of gestation (no. 1, 5), the small intestine was compressed by the uterus but could be freed by gentle traction on the intestine. In another late-term cow (no. 19), the small intestine was displaced cranially by the uterus and was trapped between the liver and gallbladder but could be repositioned manually. In one cow (no. 34), the duodenum was compressed by bands of fibrin, which could be removed. Another cow (no. 24) had adhesions between the small intestine and a 30 × 30 cm abscess in the right lower quadrant of the abdomen. The adhesions were broken down manually, and the abdomen was closed routinely. The abscess was then lanced through the right abdominal wall under ultrasound guidance, and 5 L of purulent material was drained. A common feature of all cases was that the small intestine was severely dilated proximal to the CSI and emptied distally after resolution of the compression.

In the 18 non-surviving cows (Table 5), an intra-abdominal abscess was the cause of CSI in 14 cases. In three other cows, adhesions between the small intestine and the uterus (no. 2), the omentum, abomasum, and abdominal wall (no. 14), and the liver (no. 21) could not be broken down. In another cow with hydrops allantois (no. 8), the small intestine was compressed by the dilated uterus.

Four of thirty-one (12.9%) cows had intraoperative complications consisting of intestinal rupture during manipulation in three cows (no. 4, 16, 28) and recumbency during surgery (no. 27). The three cows with intestinal rupture were euthanised intraoperatively. The cow with adhesions between the jejunum and abdominal wall (no. 20) was re-operated the next day because of no faecal output and ultrasonographic evidence of ongoing small intestinal dilatation. The affected small intestine was massaged, and the cow was discharged 4 days later and remained productive for at least 2 more years.

### 3.8. Postoperative Treatment

Medical treatment of the cows that were operated successfully and subsequently discharged included fluid therapy (17/17), antibiotics (*n* = 17/17), pain control (17/17), prokinetic drugs (12/17), and electrolyte replacement (14/17). The cows received 10 L of a solution containing 50 g glucose and 9 g sodium chloride per litre daily for 1 to 7 days (median, 95% CI, 3, 3–4 days) administered as a slow intravenous drip via an indwelling jugular vein catheter. Antibiotic treatment included penicillin G procaine (12,000 IU/kg) given intramuscularly (12/17), amoxicillin (7 mg/kg) given intramuscularly (2/17), and penicillin G procaine followed by amoxicillin (3/17). The antibiotic treatment was administered in most cases for 3 to 4 days (median, 95% CI 4, 4–4 days). All cattle received a daily injection of flunixin meglumine (1 mg/kg), ketoprofen (3 mg/kg), metamizole (35 mg/kg), or a combination of flunixin meglumine and metamizole given intravenously for 2 to 6 days (median, 95% CI, 3, 3–3 days). Prokinetic drugs were used in 12/17 cattle for a duration of 1 to 4 days (median, 95% CI, 3, 3–4 days). Seven cattle received neostigmine (40–45 mg) administered via continuous drip infusion, and five received intramuscular metoclopramide (30 mg), usually 7 to 9 times at 8 h intervals (metoclopramide was only used in the first few years). Eleven cows with hypocalcaemia received 500 mL of 40% calcium borogluconate intravenously, and ten cattle with hypokalaemia were treated with daily oral doses of 60 to 100 g of potassium chloride until normokalaemia occurred.

### 3.9. Follow-Up in 17 Surviving Cows

The general condition of 16 (94.1%) of the 17 cows that were subsequently discharged normalised within 1 to 6 days (median, 95% CI, 2, 2–4 days), the appetite of 15 cows within 1 to 8 days (3, 2–4 days) and defaecation of all cows in 1 to 8 days (median, 95% CI, 3, 2–5 days) after surgery. The rectal temperature of the 17 surviving cows did not change significantly between the time of admission (mean ± sd, 95% CI, 38.5 ± 0.5 °C, 38.3–38.6 °C) and day 7 (38.6 ± 0.33 °C, 38.4–38.9 °C). The same was true for the heart rate, which ranged from 82 ± 17 bpm (76–88 bpm) to 78 ± 12 bpm (70–87 bpm) during the same period. Thirteen (76.5%) of the seventeen cows were discharged within 4 to 8 days, and the remaining 4 (23.5%) after 12, 14, 15, and 35 days (median, 7 days).

The long-term outcome was assessed via telephone interview 2 years after discharge. Seven (41.2%) of the 17 discharged cows had remained productive in their herds. Two (11.8%) cows were slaughtered for economic reasons and four others (23.5%) for health reasons. The long-term outcome was not known for the remaining four (23.5%) cows.

### 3.10. Clinical and Laboratory Findings Concerning the Cause of CSI

The findings in the cows with CSI caused by adhesions (*n* = 16), abscesses (*n* = 16), and the pregnant uterus (*n* = 4) did not differ significantly at the time of admission, including the inflammatory variables total white blood cell count, total protein and fibrinogen concentrations, and the glutaraldehyde clotting test. There were no significant differences among the three groups for the other variables listed in Table 2.

## 4. Discussion

CSI was the least common cause of ileus in cattle seen in our clinic from 1986 to 2016. Compared with 35 cases of CSI, we treated 47 cases of small intestinal volvulus [24], 60 cases of small intestinal strangulation [25], 61 cases of mesenteric torsion [26], 85 cases of incarceration [27], and 126 cases of intussusception [19], all of which resulted in ileus. Of note, unlike other causes of ileus such as intussusception, the small intestine is not diseased per se in cattle with CSI but instead is compromised by external factors such as abscesses or adhesions that exert pressure on the intestine. This is analogous to small intestinal strangulation and incarceration of small intestinal loops within internal and external hernias.

Visceral colic is an important feature of ileus, but its prevalence in the present cows with CSI (34.3%, 12/35) was lower than in cows with small intestinal strangulation (40.0%, 24/60) [25], small intestinal intussusception (46.8%, 59/126) [19], small intestinal volvulus (66.0%, 31/47) [24], mesenteric torsion (65.6%, 40/61) [26], and ileal impaction (68.2%, 15/22) [22]. A possible explanation for this is that there is less mesenteric traction in cows with CSI and thus less stimulation of pain receptors compared with small intestinal volvulus or mesenteric torsion. Another possible reason for the absence of visceral pain in 65.7% of the cows with CSI is that the colic phase in cattle with small intestinal ileus only lasts about 12 h [20], and the cows without colic at the time of admission had already progressed to the indolence (dullness, 57.1%, 20/35) or even intoxication phase (8.6%, 3/35). Thus, it is clinically relevant that the absence of colic does not rule out ileus even though colic is considered a main symptom of ileus [21].

Similar to other causes of ileus, the principal abnormal intestinal findings were no or decreased rumen and intestinal motility, little or no production of faeces, and positive ballottement and/or percussion and simultaneous auscultation on the right side. Identification of dilated small intestines via transrectal palpation was relatively rare (25.7%, 9/35), and the actual site of compression could not be palpated. When the four cows with CSI attributable to the gravid uterus were examined, the late-term pregnancy was diagnosed but not thought to be associated with the clinical signs of ileus. Therefore, in cows pregnant for 6 months or longer with signs of ileus, uterine torsion, and CSI should be part of the differential diagnosis. Uterine torsion is diagnosed transrectally and vaginally [28], and ultrasonography may aid in the diagnosis of CSI by the gravid uterus.

Even though most cows had abscesses or adhesions caused by inflammation, the rectal temperature was increased in only five (14.3%) cows. The statement that cows with CSI and active peritonitis can have pyrexia [2] is undoubtedly correct. However, chronic inflammatory changes must not be associated with pyrexia, and thus only an increase in rectal temperature could point to CSI. This is supported by the fact that an increase in rectal temperature may also occur with small intestinal intussusception (8.0%, 10/125), mesenteric torsion (10.2%, 6/59), small intestinal volvulus (14.9%, 7/47), and small intestinal strangulation (25.0%, 15/60) (see references above).

The principal laboratory abnormality was hypochloraemic hypokalaemic metabolic alkalosis attributable to abomasal reflux, which manifested as hypokalaemia (82.9%), positive base excess (80.0%), and hypochloraemia (64.7%). This was described many years ago after experimental ligation of various regions of the small intestine [29]. An earlier study discussed the possibility of leukocytosis in cows with adhesions [2]. Based on that suggestion, we analysed the variables leukocytosis, hyperproteinaemia, hyperfibrinogenaemia, and the clotting time in the glutaraldehyde test, which are all indicative of inflammation. We were surprised to find that these variables did not differ significantly among cows with CSI caused by abscesses, adhesions, and the gravid uterus. 

Of note, the cows had a history of acute to subacute illness (with a maximum of 14 days), and chronic illness (>4 weeks) did not occur even though it can be assumed that adhesions or abscesses had developed over weeks to months and therefore were chronic. However, the observation that cows with severe lesions often do not have clinical signs, or only mild signs, for an extended period of time is not surprising. For instance, we commonly observe an apparently acute onset of illness in cows with apostematous traumatic reticuloperitonitis or large liver or reticular abscesses even though the abscesses must have been present for weeks or months. The reason for the sudden onset of overt clinical signs usually remains unknown, but it is conceivable that the lesions increase in severity over time and finally reach a stage associated with clinical manifestations. Parturition is associated with spatial changes in the abdomen and can also trigger clinical changes, for instance, when adhesions under tension cause sudden pain. On the other hand, the increasing size of the uterus in late pregnancy can lead to intestinal compression.

Intestinal adhesions caused by inflammation have been described previously [2,4,7]; they were caused by peritonitis associated with perforating abomasal or intestinal ulcers [7], omentopexy and laparotomy [7], or uterine tears [6]. One of the cows of the present study with CSI caused by adhesions (no. 27) had undergone omentopexy 10 weeks earlier, and two of three cows with adhesions originating from the uterus (no. 2, 7) calved 3 days and 5 weeks earlier. Uterine trauma was assumed to be the cause of CSI in at least cow no. 2, which had dystocia 3 days before admission, after which time she went off feed and showed mild signs of colic. In 14 Holstein cows, 8 of which had undergone right flank laparotomy for right displaced abomasum or duodenal volvulus, the reason for compression of the duodenal sigmoid flexure was adhesions involving the surgical site [7]. Tumours, and haematomas in the mesentery are considered frequent causes of CSI [1] but did not occur in this study. Compression by the gravid uterus, which occurred four times in the present study, was also mentioned in the literature as a cause of CSI [7]. A dilated gallbladder was involved in CSI in one case in the present study, but the main reason for CSI was assumed to be cranial displacement of the small intestine by the gravid uterus leading to compression of the duodenum between the liver and gallbladder. In two previous reports, CSI was caused primarily by a displaced [5] or severely dilated gallbladder [4]. Liver abscesses causing adhesions with the intestines and CSI have been described previously [3,4]. In the present study, adhesions with the liver (no. 18, 21, 23, 26, 30) or liver abscesses (no. 25) were responsible for CSI in six cases. The close anatomical proximity of the duodenum and the liver and the connection of the two organs by the hepatoduodenal ligament predispose to CSI. Fascioliasis did not occur in any of the three cows that underwent parasitological examination; however, postmortem examination showed severe dicrocoeliosis in one cow (no. 9) and severe fascioliasis in another (no. 28). After penetration of the intestinal wall, young liver flukes may cause peritonitic adhesions during their migration through the abdomen to the liver [30]. Abscesses almost always have a poor prognosis; the abdominal abscess in cow no. 24 was an exception because after breaking down the adhesions during laparotomy, the abscess could be drained transcutaneously under ultrasound guidance. Abscesses compressing the descending duodenum after right flank intraperitoneal injection [2] did not occur in this study; nevertheless, this technique should be used cautiously. We suspected that the adhesions originated from the small intestine in two cows in the present study, which may happen with an intestinal ulcer. Postmortem findings of cow no. 32 suggested a perforating jejunal foreign body as the cause of the adhesions, and cow no. 15 had a 7 cm diverticulum associated with the adhesions. Ileus attributable to compression by a jejunal diverticulum is also mentioned in another study [31], and in a 4-year-old bull, a retroflexed diverticulum in the proximal jejunum was the cause of ileus due to intussusception [32]. Finally, it should be mentioned that in our experience, based on numerous meat inspections and postmortem examinations, many cows with abdominal adhesions have no clinical signs of illness.

Laparotomy is the method of choice for diagnosing CSI in live cows. However, improvement in non-invasive diagnostic techniques, particularly ultrasonography, is needed to prevent cows with a poor prognosis from undergoing surgery. In the context of ileus, ultrasonography has mainly been used to detect dilated loops of small intestine and diminished intestinal motility, which are the principal signs of ileus. However, the underlying problem, i.e., adhesions or abscesses, could only be visualised in one cow with a large abscess. One reason for this was that most lesions were more than 15 to 20 cm away from the body surface and thus beyond the penetration depth of the sound waves. Furthermore, our examination protocol for cows with suspected ileus has until now been limited to scans from the right side and the sternal region. We think that the diagnosis could be improved with additional ultrasonography of the entire ventral abdominal wall as well as transrectally. This modified diagnostic procedure should be employed in the future and is expected to improve the diagnostic validity of ultrasonography. Hopefully, advances in ultrasound technology will also contribute to further diagnostic improvements.

It is questionable whether the intra-abdominal administration of antibiotics was justified. We originally hypothesised that the parenteral route does not provide sufficient antibiotic concentrations at the intestinal surface and suture sites as quickly as local administration. However, the analysis of three tangentially relevant reports that investigated whether parenteral or intrabdominal antibiotics are preferable concluded that when the aim is to use a product in an extralabel manner to prevent peritonitis or wound infection, postoperative intramuscular administration of sodium ampicillin or procaine penicillin G may be preferable [33].

## 5. Conclusions

Compression of the small intestine is a rare cause of ileus. Improvements in the diagnosis of CSI are needed to prevent unnecessary laparotomy in cows with a grave prognosis.

## Figures and Tables

**Table 1:** Clinical findings in cows with compression of the small intestine (means ± sd, medians, 95% CI, frequency distributions).

Variable	Finding	Number of Cows	%
Heart rate (*n* = 35, 81 ± 17 bpm, 95% CI = 75–88 bpm)	Normal (60–80 bpm)	17	48.5
	Decreased (40–59 bpm)	3	8.6
	Mildly increased (81–100 bpm)	12	34.3
	Moderately increased (101–120 bpm)	2	5.7
	Severely increased (128 bpm)	1	2.9
Rectal temperature (*n* = 35, 38.4 ± 0.5 °C, 95% CI = 38.3–38.6 °C)	Normal (38.5–39.0 °C)	15	42.9
	Decreased (37.6–38.4 °C)	15	42.9
	Increased (39.1–39.4 °C)	5	14.2
Respiratory rate (*n* = 35, median = 24 breaths per min, 95% CI = 20–28 breaths per min)	Normal (15–25 breaths per min)	18	51.4
	Decreased (12–14 breaths per min)	2	5.7
	Increased (26–60 breaths per min)	15	42.9
Rumen motility (*n* = 35)	Decreased	26	74.3
	Absent	9	25.7
Foreign body tests (*n* = 35)	All negative	22	62.9
	At least one test positive ^1^	9	25.7
	Equivocal	4	11.4
BSA and PSA on the left side (*n* = 34)	Both tests negative (normal)	33	97.1
	Both tests positive	1	2.9
BSA and PSA on the right side (*n* = 34)	Both tests negative (normal)	9	26.5
	Only BSA positive	12	35.3
	Only PSA positive	3	8.8
	Both tests positive	10	29.4
Intestinal motility (*n* = 35)	Normal	5	14.3
	Decreased	22	62.8
	Absent	8	22.9
Rectal findings ^2^ (*n* = 35)	Normal findings	24	68.6
	Rumen dilated	10	28.6
	Dilated loops of small intestines	9	25.7
	Dilatation of the caecum and/or spiral colon	5	14.3
	Taut tissue bands	1	2.9
Faeces, amount (*n* = 35)	Faecal output reduced	25	71.4
	No faecal output	10	28.6
Faeces, degree of comminution (*n* = 32)	Normal (well digested)	18	56.2
	Moderately digested	2	6.3
	Poorly digested	2	6.3
	No faecal output	10	31.2
Faeces, consistency (*n* = 35)	Normal (pulpy)	11	31.4
	Thin pulpy	5	14.3
	Thick pulpy	2	5.7
	Liquid	1	2.9
	Biphasic	2	5.7
	Pasty	4	11.4
	No faecal output	10	28.6
Faeces, colour and abnormal contents in the rectum ^3^ (*n* = 35)	Normal (olive)	21	61.1
	Dark (brown to black)	2	5.6
	Mucus	8	22.2
	Blood	4	11.1
	Fibrin	2	5.6
	Several abnormal contents	2	5.6
	No faecal output	10	27.8

^1^ Positive: at least 3 of 4 tests elicited a grunt. ^2^ The total number of findings was 49 (140.1%) because 14 cattle had more than one abnormal transrectal finding. ^3^ The total number of findings was 49 (139.0%) because 14 cattle had more than one abnormal finding. BSA—ballottement and simultaneous auscultation. PSA—percussion and simultaneous auscultation.

**Table 2:** Laboratory findings in cows with compression of the small intestine (means, medians, standard deviations, 95% CI, frequency distributions).

Variable (Mean ± sd, Median, 95% CI)	Finding	Number of Cows	Percent
Haematocrit (*n* = 35), 36.0 ± 5.6%, 95% CI = 34–38%	Normal (30–35%) ^1^	16	45.7
	Decreased (26–29%)	3	8.6
	Increased (36–50%)	16	45.7
Leukocytes (*n* = 35), 9.745 ± 3362/µL, 95% CI = 8.591–10.901/µL	Normal (5.000–10.000/µL)	17	48.6
	Decreased (3.800–4.999/µL)	3	8.6
	Increased (10.001–17.000/µL)	15	42.8
Total protein (*n* = 35), 86.0 ± 9.9 g/L, 95% CI = 83–89 g/L	Normal (60–80 g/L)	13	37.1
	Increased (81–118 g/L)	22	62.9
Fibrinogen (*n* = 35), 7.6 ± 2.4 g/L, 95% CI = 6.8–8.4 g/L	Normal (4–7 g/L)	14	40
	Decreased (3.0–3.9 g/L)	1	2.9
	Increased (7.1–14.0 g/L)	20	57.1
Urea (*n* = 35), 9.8 mmol/L, 95% CI = 6.0–11.5 mmol/L	Normal (3.3–6.5 mmol/L)	13	37.1
	Increased (6.6–24.7 mmol/L)	22	62.9
Bilirubin (*n* = 35), 5.6 µmol/L, 95% CI = 3.7–7.2 µmol/L	Normal (1.3–6.5 µmol/L)	23	65.7
	Increased (6.6–26.9 µmol/L)	12	34.3
Calcium (*n* = 22), 2.13 ± 0.31 mmol/L, 95% CI = 1.99–2.27 mmol/L	Normal (2.30–2.60 mmol/L)	7	31.8
	Decreased (1.36–2.29 mmol/L)	15	68.2
Magnesium (*n* = 20), 1.22 ± 0.31 mmol/L, 95% CI = 1.09–1.35 mmol/L	Normal (0.80–1.00 mmol/L)	5	25
	Increased (1.01–1.69 mmol/L)	15	75
Inorg. phosphate (*n* = 21), 1.69 ± 0.63 mmol/L, 95% CI = 1.40–1.98 mmol/L	Normal (1.30–2.40 mmol/L)	14	66.7
	Decreased (0.37–1.29 mmol/L)	4	19
	Increased (2.41–3.25 mmol/L)	3	14.3
Chloride (*n* = 34), 89 ± 13 mmol/L, 95% CI = 85–94 mmol/L	Normal (96–105 mmol/L)	8	23.5
	Decreased (65–95 mmol/L)	22	64.7
	Increased (106–116 mmol/L)	4	11.8
Potassium (*n* = 35), 2.90 mmol/L, 95% CI = 2.6–3.4 mmol/L	Normal (4.0–5.0 mmol/L)	5	14.3
	Decreased (1.8–3.9 mmol/L)	29	82.9
	Increased (6.5 mmol/L)	1	2.8
AST (*n* = 35), 83 U/L, 95% CI = 71–96 U/L	Normal (25–103 U/L)	26	74.3
	Increased (104–144 U/L)	9	25.7
γ-GT (*n* = 35), 22 U/L, 95% CI = 19–25 U/L	Normal (13–30 U/L)	28	80
	Increased (31–52 U/L)	7	20
pH (*n* = 30), 7.45 ± 0.06, 95% CI = 7.43–7.47	Normal (7.41–7.45)	9	30
	Decreased (7.31–7.40)	7	23.3
	Increased (7.46–7.54)	14	46.7
pCO_2_ (*n* = 30), 50.1 ± 9.43 mmHg, 95% CI = 47–54 mmHg	Normal (35.0–45.0 mmHg)	10	33.3
	Decreased (33.4–34.9 mmHg)	1	3.3
	Increased (45.1–69.0 mmHg)	19	63.4
Bicarbonate (*n* = 30), 31.3 mmol/L, 95% CI = 27–36 mmol/L	Normal (20.0–30.0 mmol/L)	14	46.7
	Increased (30.1–52.5 mmol/L)	16	53.3
Base Excess (*n* = 30), 9.5 mmol/L, 95% CI = 6.5–13.0 mmol/L	Normal (−2–+2 mmol/L)	3	10
	Decreased (−3.7–−2.1 mmol/L)	3	10
	Increased (+2.1–+27.4 mmol/L)	24	80
Glutaraldehyde test (*n* = 32), 7.0 min, 95% CI = 5.0–10.0 min)	Normal (≥7 min)	15	46.9
	Shortened (<7 min)	17	53.1
Rumen chloride (*n* = 34), 25.5 mmol/L, 95% CI = 21–30 mmol/L	Normal (≤30 mmol/L)	24	70.6
	Increased (31–104 mmol/L)	10	29.4

^1^ Reference [17] for the normal range of all variables.

**Table 3:** Ultrasonographic findings in cows with compression of the small intestine.

Variable	Finding	Number of Cows	%
Intestinal motility (*n* = 20)	Subjectively decreased	8	40
	Absent	12	60
Cross-section of small intestine (*n* = 23)	Normal (3.5 cm) ^1^	2	8.7
	Dilated (4.0–10.0 cm)	21	91.3
Free fluid in the abdomen (*n* = 23)	No fluid visible	14	60.9
	Fluid without fibrin	3	13
	Fluid with fibrin	6	26.1
Fibrin between the intestines (*n* = 23)	No fibrin visible	19	82.6
	Fibrin visible	4	17.4
Empty loops of poststenotic small intestines (*n* = 23)	Not visible	17	73.9
	Visible	6	26.1
Compression visible (*n* = 23)	Not visible	22	95.7
	Visible ^2^	1	4.3
Abomasal dilatation (*n* = 23)	Not dilated	18	78.3
	Dilated	5	21.7

^1^ Reference no. 12 for the normal range of cross-section of the small intestine. ^2^ Adhesions between dilated jejunum and chambered abdominal abscess (no. 35).

**Table 4:** Surgical findings and surgical treatment in 17 surviving cows with compression of the small intestine (the cases are listed in chronological order from 1986 to 2016).

Case No.	Description	Surgical Findings	Surgical Treatment
1	Brown Swiss, 4.0 yrs	Small intestine trapped and compressed by gravid uterus (7 months pregnant)	Small intestine repositioned
5	Holstein, 7.5 yrs	Small intestine trapped and compressed between gravid uterus (6 months pregnant) and abdominal wall	Small intestine repositioned
6	Brown Swiss, 4.0 yrs	Adhesions between small intestine and omasum; cow 9 months and 10 days pregnant	Adhesions only partially broken down; induction of labour
10	Brown Swiss, 4.5 yrs	Adhesions between distal jejunum and urinary bladder (uterine infusion 6 weeks earlier after birth of twins)	Adhesions broken down manually
12	Brown Swiss, 3.0 yrs	Adhesions between right uterine horn and abdominal wall and jejunum	Resection of 30 cm of jejunum because of focal necrosis of intestinal wall at adhesion site; no information about previous labour
15	Brown Swiss, 4.0 yrs	7 cm diverticulum of the jejunum with adhesions involving the greater omentum	Resection of 40 cm of jejunum
18	Brown Swiss, 3.5 yrs	Adhesions between reticulum, liver, peritoneum, greater omentum and jejunum	8 cm adhesion between jejunum and greater omentum broken down manually
19	Brown Swiss, 5.0 yrs	Small intestine displaced cranially by gravid uterus (9 months pregnant) and duodenum trapped between liver and gallbladder	Repositioning of duodenum
20	Brown Swiss, 4.5 yrs	Adhesions between jejunum and abdominal wall caused by necrosis of intestinal wall	Adhesions broken down manually and resection of the affected intestine. Second surgery one day later because of intestinal paralysis; treated with intestinal massage
23	Brown Swiss, 5.0 yrs	3 cm × 3 cm adhesion between duodenum and liver	Adhesions broken down manually
24	Brown Swiss, 3.7 yrs	30 cm × 30 cm abdominal abscess in the lower right quadrant with adhesions to jejunum	Adhesion between abscess and jejunum broken down manually. Abscess lanced under ultrasonographic guidance from the right abdominal wall and 5 L of purulent material drained
26	Holstein, 8.9 yrs	Two focal adhesions between jejunum and liver	Adhesions broken down manually
27	Swiss Fleckvieh, 6.4 yrs	Adhesions between the ascending duodenum and the omentopexy site after abomasal surgery 10 weeks previously	Omentopexy and duodenal adhesions broken down manually
30	Brown Swiss, 2.3 yrs	Multiple adhesions between the sigmoid flexure of the duodenum and the liver	Most adhesions broken down manually
31	Swiss Fleckvieh, 11 yrs	Adhesions between duodenal sigmoid flexure and lesser omentum	Adhesions broken down manually
33	Brown Swiss, 3.2 yrs	Adhesions between two distant jejunal loops because of bowel rupture	Adhesions broken down manually and resection of the perforated intestine
34	Brown Swiss, 2.8 yrs	Caudal duodenal flexure compressed by fibrin masses	Removal of fibrin masses and oversewing of the duodenal serosa

**Table 5:** Surgical and postmortem findings in 18 non-surviving cows with small intestinal compression (the cases are listed in chronological order from 1986 to 2016).

Case No.	Description	Surgical Findings	Postmortem Findings
2	Brown Swiss, 2.5 yrs	Adhesions between jejunum and uterus	Adhesions between small intestine and uterus, peritonitis, multiple abomasal ulcers (the cow had dystocia 3 days before admission together with colic and anorexia)
3	Brown Swiss, 5 yrs	Adhesions between small intestine and intra-abdominal abscess	Large adhesions between small intestine and intra-abdominal abscess, peritonitis
4	Swiss Fleckvieh, 3 yrs	Adhesions between small and large intestines, bowel rupture during attempt at exteriorisation of the intestine	Adhesions between jejunum and spiral colon involving a 12 cm abscess
7	Brown Swiss, 3 yrs	Adhesions between small intestine and uterine abscess	Uterine abscess involving the greater omentum, mesentery and small intestine (5 weeks after parturition)
8	Brown Swiss, 4 yrs	Slaughtered immediately after clinical examination	Hydrops allantois, 9 months pregnant, compression of jejunum by uterus
9	Swiss Fleckvieh, 6 yrs	Severe adhesions involving the small intestine that could not be broken down	Abscess between small intestine and spiral colon compressing the small intestine; reticulum, rumen and liver also involved in the adhesions; severe dicrocoeliosis
11	Brown Swiss, 5 yrs	Slaughtered because owner did not consent to surgery	Abscess in the mesojejunum compressing the small intestine; necrotising jejunitis, abomasal ulcer, fatty liver
13	Brown Swiss, 12 yrs	Adhesions between abdominal wall, greater omentum, small intestine and omasum	Abscess in the mesojejunum compressing the small intestine; adhesions
14	Holstein, 6 yrs	Adhesions between greater omentum, abdominal wall and small intestine compressing the small intestine	Adhesions between greater omentum, abomasum, abdominal wall and small intestine compressing the small intestine
16	Brown Swiss, 3.5 yrs	Purulent peritonitis with severe apostematous adhesions of the small intestine; bowel rupture during attempt at exteriorisation of the intestine	Purulent peritonitis and adhesions involving the reticulum, rumen, omasum, abomasum and small intestine with abscessation and compression of the small intestine
17	Brown Swiss, 7 yrs	Slaughtered because owner did not consent to surgery	Adhesions between small intestinal loops that could not be broken down and several abscesses up to 2 cm in diameter in the small intestinal wall compressing the wall
21	Brown Swiss, 3 yrs	Adhesions between small intestine and liver	Adhesions between small intestine and liver and compression of the small intestine
22	Brown Swiss, 3 yrs	Adhesions between small intestinal loops that could not be broken down	Adhesions between small intestine and spiral colon; abscess and fat necrosis in the mesocolon
25	Brown Swiss, 7.6 yrs cow	Adhesions between small intestine and liver abscess that could not be broken down	Adhesions between liver and duodenum and jejunum and compression of small intestine
28	Brown Swiss, 6.3 yrs	Severe adhesions between distal jejunal loops. Bowel rupture during attempt at exteriorisation of the intestine	Focal fibrinous peritonitis involving the distal jejunum with adhesions that could not be broken down; reticular abscess; severe fascioliasis
29	Brown Swiss, 2.8 yrs	Adhesions between small intestine and abdominal wall that could not be broken down or exteriorised	Adhesions between small intestine and intra-abdominal abscess and compression of the small intestine
32	Holstein, 3.5 yrs	Adhesions between two loops of small intestine that could not be broken down	Jejunal abscess and adhesions between jejunal loops and gall bladder, possibly caused by perforating intestinal foreign body
35	Brown Swiss, 8 yrs	Euthanasia based on severe ultrasonographic changes (adhesions between large intra-abdominal abscess and small intestine)	Adhesions between jejunum, ileum and intra-abdominal abscess

## Data Availability

The datasets used and analysed for this study are available from the corresponding author on reasonable request.
